# How to Do Things With Texts: A Functional Account of Reading Comprehension

**DOI:** 10.1007/s40616-020-00135-0

**Published:** 2020-12-07

**Authors:** Eileen Pfeiffer Flores, Jorge Mendes de Oliveira-Castro, Carlos Barbosa Alves de Souza

**Affiliations:** 1 King’s College, London, King’s College, Philosophy Building, Strand Campus, London, WC2R 2ND UK; 2 University of Brasilia, Department of Basic Psychological Processes, Psychology Institute – ICC SUL – Campus Darcy Ribeiro, Asa Norte, CEP 70 910900 Brasília, DF Brazil; 3 Federal University of Pará, Belém, Pará Brazil

**Keywords:** Comprehension, Legal texts, Narrative, Reading, Text

## Abstract

We offer an account of reading comprehension that we believe will help clarify some common conceptual confusions in the relevant literature, as well as contribute to existing functional accounts. We argue that defining texts qua texts as stimulus classes, on the one hand, and equating “comprehension” with behavior (covert or otherwise), on the other, are not useful conceptual moves, especially when behavioral settings go beyond basic literacy skills acquisition. We then analyze the structure of the contingencies that usually evoke talk of “comprehension” using techniques from analytic philosophy. We show how keeping the results of this analysis in mind can help avoid the conceptual bafflement that often arises, even among behavior analysts, when defining or assessing behavioral phenomena related to reading comprehension. Using two contrasting cases (legal texts and stories), we argue that what counts as comprehension depends, not peripherally but crucially, on the shared social practices of which texts are a part. Finally, we propose a new framework for classifying reader–text contingencies by combining two dimensions: openness of setting and embeddedness of reinforcement.

In this article, we offer a functional analysis of what can be broadly characterized as “reading comprehension,”^1^ which we believe will enrich existing theoretical accounts, as well as clarify some common conceptual confusions in the field. The outline of the article is as follows: First, we will defend that defining the text qua text as a class of stimuli (i.e., treating texts as fairly univocal in their effects upon readers) is not always useful when investigating comprehension (more on this in the course of our argument). As we will see, this is especially true when behavioral settings go beyond the simple one-to-one correspondence between text and behavior that is typical of basic literacy skills acquisition.

A second, complementary argument is that, beyond textual repertoires, it may not be advantageous to define reading comprehension as a class of behaviors, private or otherwise. In order to support this claim, we carry out an analysis that uncovers the structure of the contingencies that usually lead us to speak of “comprehension.” As we will show, these contingencies are not readily discriminable when we use the concept in everyday and even technical contexts, thus tempting us to conceive of comprehension as a private event or chain of events that accompanies overt behavior.

The characteristic features of the contingencies usually at work when we reinforce talk of comprehension (and other related terms, such as “understanding” and “grasping”) can be conveyed by the notion of the “logical structure of the concept of comprehension,” which is our chosen way of speaking in this article. There are two reasons for this choice. First, it is more concise. To give an example unrelated to this article, when one says that the concept of religiosity is (usually) predicated only to human beings, this is a concise and convenient way of summarizing the fact that the practices of our verbal community do not, in general, include the reinforcement of the generalization of talk of religiosity from human contexts to ones involving nonhuman animals or inanimate objects. Second, our choice allows us to draw on useful technical terms already developed in philosophy, such as the notions of dispositional, adverbial, and polymorphous features of concepts (to be explained later). None of this risks the reification of concepts so long as we remember that, when we summarize contingencies by speaking of the logical features of the concept *x*, we are not bringing in an explanatory entity, but only employing a handy technical terminology that helps bring out regularities in our lived interactions.

Our analysis of comprehension will be followed by the application of our reasoning to two different kinds of texts: legal documents and narratives. Finally, we will propose a preliminary integrative model, which we hope may be useful when investigating or assessing reading comprehension in various settings.

## Texts as Discriminative Stimuli

Texts may function as discriminative stimuli for relatively uniform responses for different readers, for example, in the acquisition and maintenance of the formal relations of stimulus control known as textual behavior (Skinner, 1957). In order to acquire discriminative functions, the verbal stimuli must have been accompanied by a history of differential reinforcement. Thus, a text’s role as a discriminative stimulus usually involves a long and intricate learning process. To illustrate this, think of a passage in Japanese shown to someone who has never learned the language. In this case, the written characters do not increase the probability of vocal responses that anyone would call “reading” (although they may induce responses such as “I cannot read Japanese.”). In sum, characters on a page become discriminative through a painstaking process of differential reinforcement by the verbal community.

One could take the fact that every reader has a different history of learning to entail that a given text would have as many meanings or interpretations as there are readers. The latter, in turn, might lead to the conclusion that there is no limit to possible interpretations of a text, which seems absurd. What bridges the gap between the facts that, on the one hand, each person has a unique history of reinforcement and, on the other, we speak meaningfully about the texts we share is that the learning experiences of different readers arise from general social practices concerning what are considered to be correct or adequate responses in the presence of given texts.

Some of the social reinforcement contingencies for reading behaviors are very specific and homogeneous. Take, for instance, those involved in teaching essential literacy skills. The verbal community’s reinforcement practices are relatively straightforward when shaping a child’s responses to syllables or individual words. Reinforcement contingencies are characterized as specific because there are programmed consequences for a specific range of sounds emitted by the child. They are said to be homogeneous because similar text–sound relations are followed by similar consequences in most contexts in a given verbal community. As the child acquires textual repertoires and the complexity of the material increases (e.g., reading a storybook), other verbal responses beyond simple point-to-point textual relations become more and more prevalent, and reinforcement contingencies tend to become less specific and less homogeneous, varying across verbal contexts. With time, they also branch out into the reinforcement practices typical of specialized activities, where they become attuned to the specific purposes of texts as nested in those activities.

To recapitulate, as we drift from the more specific point-to-point discriminative relations to the relations of thematic control^2^ (Skinner, 1957) that are present in more complex texts, responses considered to be correct or adequate become (a) less homogeneous and (b) more and more dependent on the specific human activities in which reading is embedded. When examining more complex reader–text relationships, the standard conceptualization of texts as discriminative stimuli may not capture all that is involved. We do not mean that books, documents, and the like cease to be stimuli in the environment, but that texts qua texts do not constitute a class of stimuli in most situations. In other words, their functions in each setting cannot be fixed by looking merely at the fact that they are texts. Consequently, generic definitions of texts, for example, as descriptions of contingencies, although not necessarily incorrect, do not do enough work toward helping us understand their functions in a given network of contingencies (and may mislead us into thinking that there *must* be a common function to everything that we call a “text”).

An example may serve to illustrate the points made so far. We can describe the functions of legal texts generically as discriminative stimuli, in the sense that they can signal consequences for someone’s behavior (especially in a statutory legal system). For example, a statute might indicate that a given sentence or decision (the judge’s behavior) has a high probability of being reversed at a higher instance (probably an aversive consequence for the judge’s action). This same statute presumably signals something different to a defense attorney, such as that quoting it during the trial might lead to a reduction in the harshness of the sentence (i.e., that quoting it will be reinforced). In other words, the same written material performs different functions, depending on the context and on motivational variables.

However, this generic interpretation of the text as an object that signals consequences, as a green light in an operant box might do, is only partly appropriate. In one sense, the light and statute are equivalent. Just as the green light only works as a discriminative stimulus because of its previous association with reinforcing consequences, the statute’s signaling this or that consequence also depends on an associative history. In the case of the statute, however, it is not merely a matter of presence or absence. We must somehow account for how our behavior relates to what is sometimes called the content of written material—in other words, what it means to say we understand a given text. Before we do this, let us look at some other aspects of texts as stimuli.

### Transfer of Functions Among Stimuli

A central example of the approach to texts as stimuli is the literature on stimulus equivalence (e.g., Sidman, 1994). Studies in this domain have explored some aspects of reading comprehension by demonstrating that different stimuli can become functionally equivalent in (some of) their response-inducing properties after a history of pairings (e.g., through matching-to-sample procedures; cf. Sidman, 1994). What was new in contrast with the older paired-associate literature was that these studies demonstrated that it was not necessary to pair all the members of a set of stimuli among themselves for them to acquire similar behavior-inducing properties. For example, teaching to pair stimuli A and B, then stimuli B and C, might suffice for A and C to become functionally equivalent for some behaviors, even though their pairing had never been reinforced. The classical experimental model of stimulus equivalence usually involves teaching the subject a matching-to-sample task—that is, choosing a comparison stimulus in the presence of a sample. For instance, a child learns to point to a picture of a duck (B) when the sample is a vocal stimulus “duck” (A) and learns to point to the same image (B) when the model is the written word “duck” (C). She has thus learned two relations: A-B and A-C. Without any specific training, it is often the case that she can point to the word “duck” (C) upon seeing the picture (B). Thus, from two trained pairings (A-B and A-C), a derived pairing is also learned: B-C.

Of course, things are much less straightforward when we abandon strict adherence to the presentation of one single model at a time and to pointing (or clicking) as the standard response in all cases. The inconspicuous and uniform nature of the responses used in classic equivalence experiments (pointing, clicking) may be one of the factors that have made the idea of stimulus–stimulus relations so appealing. In any case, the literature on stimulus equivalence points to one of the ways in which the behavioral functions of one stimulus can come to be exerted by another stimulus in the absence of direct training. Beyond relations of formal control, however, the model works less well for the purposes of analyzing reading comprehension, because of its strong anchor in a referential picture of language (a topic beyond the scope of this article and already covered by other authors, e.g., Tonneau, 2001).^3^

### Higher Order Behavior and *As-If* Patterns

Another relevant way of obtaining the transfer of stimulus function is the one involved in the emergence of some kinds of higher order behavioral sequences (Oliveira-Castro, Faria, Dias, & Coelho, 2002). For example, let us say Mary memorizes her bank account’s personal identification number (PIN). The sequence might go as follows: First, access to her new bank account becomes reinforcing because Mary needs to check her balance for some purpose (i.e., a motivational operation is present). Second, Mary checks her PIN on the letter she has received from the bank (a precurrent response). Third, she types in her PIN. Fourth, she accesses her bank account (this is the reinforcing event for the entire response chain). After a few trials, discriminative functions are generalized from the PIN on the letter to the sign-in page (or to the name of the bank, its logo, etc.). Mary now types in the PIN as soon as she opens the application, without needing to look it up. That is, the precurrent response is no longer required. In ordinary language, we say Mary has memorized her PIN. In technical terms, there has been a transfer of the stimulus function from the PIN printed on the letter to the start page in the application.

Why this happens probably has to do with a reduction in the response cost and delay to reinforcement (cf. Oliveira-Castro, 2003; Oliveira-Castro et al., 2002). Because the precurrent response of looking up the PIN is not necessary for reinforcement delivery, it tends to decrease when the transfer of stimulus function is possible and can lead to faster access to reinforcement (cf. Oliveira-Castro, 2000). Thus, a higher order behavioral unit emerges. In the PIN example, the behavioral unit *opening the sign-in page in the application—looking up the pin—typing in the pin* becomes the shorter sequence *opening the sign-in page—typing in the PIN*. The latter is a higher order response because it derives from a history of learning involving the former.

From a representationalist stance (cf. Crane, 2015, for a synthesis of this point of view), this would be described as Mary now having an internal representation of her PIN. From a functional-analytic standpoint, however, there is no need to posit internal duplicates of contingencies or parts of contingencies (Skinner, 1969/2013). Mary does not continue to look up her PIN in her head—she simply stops looking it up. To say the number is now in her mind is precisely to say it is not anywhere (although many neuroscientists do think it is somewhere when they search for representations in the brain, e.g., as correlations between patterns of neural activity and stimuli).^4^ Saying the PIN is in Mary’s memory or in her mind is an expression that has a negative function (cf. Ryle, 1949/2009), in that it expresses precisely that Mary *no longer* needs to go through some of the steps in the chain (not even covertly) in order for her behavior to produce reinforcement.

Let us take another look at the stimulus equivalence paradigm. The more general idea behind equivalence relations, which it has in common with the formation of higher order sequences as just described, is that stimuli may, in some contexts, come to have similar functions, even without their being, in themselves, physically similar. A photographic depiction of a duck, therefore, carries some of the inducing functions of a real duck. However, what does it mean to say that events *A* and *B* have similar functions? It seems clear that the effect of the picture on verbal behavior can vary and is not limited to occasioning the utterance of the word “duck” (e.g., “Gray and white,” or “Very small. I think it is a duckling.”). The picture allows the person to do some things as if the duck were present.

But the person does not see a duck; she sees a photograph of a duck. Many aspects present in the context of there-being-a-duck are naturally not present in the context of the picture, such as noise, temperature, odors, or motion. Thus, there is programmed reinforcement for some things the person would do in the presence of a real duck, such as describing its size and color, but not for all of them (some are not plausible, and others are not even possible). In some senses, it is as if she could see a duck. The same applies to our formal example of memorizing a PIN. The emergent higher order pattern might lead Mary to say it is *as if* she could see the number. This means she can do some things as if she were looking at it (e.g., type it in), but not all of them (if she forgets her PIN again, she obviously cannot resort to looking up the number “in her mind’s eye”).

This *as-if* pattern of some forms of higher order behavior can also be present when we are reading, which approximates it, in some respects, to pretending (more on this later). When someone pretends, for example, to be a bear while playing with a child, the person pretending to be a bear does things that (she thinks) bears typically do. She may walk on all fours, maybe growl, stand up, and open her arms, playfully pretending to “attack.” The point here is that she must have learned something about bears in order to pretend to be one, be it in nature, at the zoo, in movies, from books, or in cartoons. Pretending to be a bear—that is, acting as if she were a bear—is a higher order behavioral pattern, dependent on acquaintance with how real bears (or cartoon bears—the imitation may be several degrees away from any original model) behave. Similarly, a child might pretend to be throwing a birthday party for one of her stuffed animals, but this is only possible if she already has some acquaintance with birthday parties (cf. Austin & Anscombe, 1958). We will have the opportunity to revisit these *as-if* patterns of behavior later, when we discuss legal texts and narratives.

## A Functional Approach to Reading Comprehension

### Text Comprehension Is Not a Private Event, Nor an Operant Class

Outside the behavior-analytic tradition, scholars often take it for granted that text comprehension is an unobservable process or event. Pearson (2009), for example, stated, in the introduction to a compilation of empirical research,

Comprehension, or “understanding”, by its very nature, is a phenomenon that can only be observed *indirectly*. . . . We talk about the “click” of comprehension that propels a reader through a text, yet we never see it. We can only rely on indirect symptoms and artifacts of its occurrence. (p. 3; cf. also, more recently, Pearson & Cervetti, 2017, where the authors sustained and reemphasized this same conception)

What is it that makes understanding so mysterious? Is it that it is a covert event? Is this covert event a kind of sudden “click”? It is surely commonplace that someone understands a text, can recall it and elaborate on it, yet has not had any experience of such a “click.” Does that mean that she did not really understand it? We probably would not conclude this. There is something odd in considering understanding as a hidden occurrence, yet it is a pervasive notion.

The idea that we must content ourselves with indirect measures of comprehension (Pearson & Cervetti, 2017) stems from the belief that we can only access symptoms or signs of understanding. Understanding itself can never be accessed directly, according to this widespread conception. However, let us suppose someone can tell the story she has just read, elaborate on it, retell it in different ways without altering its general sense, show awareness of the gist of the story’s plot, and so on. Will we still feel inclined to say that we do not really know if she understands the story because, after all, we only have access to symptoms of understanding, but not to comprehension itself? The conclusion seems absurd, which indicates that comprehension is perhaps not something else that lies beyond these behaviors, but a concept that summarizes them.

Is comprehension then a class of responses? This possible solution also baffles us as soon as we try to unravel it. For what would unify the different responses under this class, except the fact that we call them all comprehension? An operant class is a class of behaviors that produce similar consequences—in other words, a set of responses that change the environment in similar ways. If a response *r* belongs to an operant class *O*, the reinforcement or punishment of any other response in *O* shall also affect *r*. This reasoning does not seem to apply to comprehension at all, not least because it is not clear what could count as “comprehending” every possible text. Even telling someone else what the text says “using one’s own words” does not apply in all cases. It fits the situation of a teacher testing whether a child understands a paragraph in a history book, but not that of following a set of instructions in a recipe. In the second situation, following instructions aptly is much more indicative of comprehension than paraphrasing them. What would be the common consequences of the two? They seem to be very different: the teacher’s approval and maybe a good grade in one case, and baking and eating a lovely cake in the other. And what do both, in turn, have in common with the supposed “consequences of understanding” Virginia Woolf’s *To the Lighthouse*?

A possible answer to this question comes from recognizing that behaviors that count as “comprehending” and their consequences are different depending on the circumstances. This conclusion is what we are arguing for and takes us back to our initial statement: “Comprehension” is not a class of responses. The concept of comprehension functions as what Bennett and Hacker (2003) called a “polymorphous concept”—that is, the criteria for saying “she understands” might have nothing in common from one situation to the next (compare striking a particular note at the right time when reading a score with feeling antipathy toward a character when reading a novel).

### Dispositional and Adverbial Aspects of the Concept of Comprehension

The reason it is misleading to reduce comprehension to a response class is that comprehending is not behavior at all (although it involves behavior). The fact that it does not name a response class, however, does not commit us to conclude that it must therefore be a private event or a mysterious covert “click.”

Comprehension is a concept with several complementary characteristics, all of which are important for making sense of it. Therefore, as we list and explain its features, the reader should consider that none of them exhausts, on its own, the concept’s functioning. As we describe them one by one, the pieces will gradually fall into place, and we will arrive at an “overview” of the concept’s operation. Hopefully, this will help us resist the temptation to try to pigeonhole it into being either an event or a kind of behavior.

#### Dispositional features

Comprehension (when reading, but also comprehension of an idea, of a concept, or of the workings of a machine) functions, in many contexts, as what Ryle (1949/2009) called a dispositional concept. When we say that someone understands, for example, a scientific journal article, we are not pointing to, nor naming, some event or behavior. It is not the name of an occurrence. Instead, we are affirming that we expect the person to be able to do certain things with what she has read from that text (none of which she needs to be doing at that moment).

What we expect the reader to be capable of depends on the context and the degree of expertise we require. If a graduate student claims to understand an article, we might expect her to be able to say what the article is about and how the argument is structured, and to describe the evidence. We might also expect her to discern what is most relevant in the study (in contrast to dutifully listing off every detail). In more advanced settings, we sometimes request that the reader detect flaws in reasoning or in how the data are analyzed, or relate the study to a broader field of knowledge. As we can see, the things we expect—that is, arrangements of social reinforcement—depend on the kind of human activity that the text is a part of (more on this later). The emphasis here is on the fact that the concept functions as a kind of synthesis of if-then relations. For example, if asked to describe the gist of the paper, the reader does so competently; if there is a blatant incoherence between a graph and its description, she recognizes it, and so on.

The latter are not outward manifestations, signs, or symptoms of the comprehension of an article; on the contrary, they are what we mean when we say that someone comprehends an article. Evidence of this is that if the student could not string two coherent sentences together about the article, we would probably conclude that she did not understand the text very well or even at all. In order to reach this conclusion, of course, it would be necessary to exclude any factors irrelevant to our point, such as shyness, temporary states such as drowsiness or drunkenness, deliberately pretending not to understand (e.g., for fear of seeming arrogant or pedantic), and so on. Then, if we could exclude such possibilities (or had no reason to suspect them) and had tried several ways of bringing out the student’s grasp of the text, with no results, we would probably maintain the conclusion of her not having understood the text. What we would probably *not* conclude is that we will never really know whether anybody has comprehended anything, a conclusion based on the assumption that being able to do things such as talk about the text are only “outward manifestations” of comprehension. Such an interpretation would eliminate all meaning from the concept. The verbal response of saying that someone has comprehended a given text would perform no function in social interactions.

So far, we have described the dispositional property of comprehension. We have seen that the concept synthesizes a set of if-then clauses, the limits of which may be more or less flexible (see also Alvarez, 2014; for a discussion of the flexibility or “openness” of many dispositional concepts, see Ryle, 1949/2009). For example, if Bianca is said to understand an algebraic formula, this sums up possibilities of if-then clauses such as “if confronted with a problem for which the formula is relevant, she uses it appropriately,” “if asked to explain the formula, she gives an intelligible explanation,” and “if asked to point out an error, she will at least work toward this in a coherent way.” It is noteworthy that (a) anyone judging Bianca’s comprehension of the formula must also understand it, and (b) some manifestations of comprehension are more central than others. For example, being able to use the formula to solve different problems is more central than being able to explain it to someone else. If Bianca is capable of the former but not the latter, the verbal community will usually accept that she understands the formula, whereas the opposite seems unlikely and, if it happens, may raise doubts regarding rote memorization.

It is also important to remember that comprehension can have degrees and nuances. We can understand a novel better upon a second reading than upon the first, understand it on some levels but not others, understand it on a different level after studying literary theory or going through significant personal experience, and the like.

The previous point seems at first sight to contradict what we claim next—that is, that comprehension (in this case, “to comprehend”) is an achievement verb. However, we hope to show that the fact that comprehension can have nuances and degrees is orthogonal to the fact that it describes results and not attempts—in other words, that it belongs to the broader family of achievement verbs. First, let us explain what achievement verbs are. Ryle (1949/2009) pointed out that some verbs do not apply to actions, but rather to the results of our actions. A typical example is the verb “to cure.” We can say that a doctor treated someone but has failed to cure her, but if the doctor said she had cured the patient, but unfortunately the patient had not gotten any better, we would probably be confused as to what she meant. For “to cure” does not indicate an action nor a series of actions. It summarizes a result of actions (a positive one—as a result of our actions, someone who was ill is now healthy). Teaching is arguably also an example of this kind, although, strangely, some teachers do sometimes say their students are not learning what they teach them (this sometimes raises the objection that, in this case, no teaching has taken place at all, but only the attempt to do so). The ambiguity in the case of teaching probably comes from the fact that the concept is also a general term for teachers’ activities (somewhat like “gardening” or “doing bricolage”). Perhaps closer to “comprehending” is “learning” or “healing,” in that they refer to a result regarding ourselves and not another. We hope the reader can apply the previous reasoning to these two last examples and see that they indicate achievements or results, not actions. The same applies to comprehension. In sum, contrary to what researchers often take for granted (e.g., Kendeou, Van Den Broek, Helder, & Karlsson, 2014; Madau, 2017; Pearson & Cervetti, 2017), comprehension is not an ongoing inner process resulting in something else. Instead, it is the result of actions and activities, such as studying, reading, discussing, or asking questions, just as healing is a result of, for example, resting, eating the right foods, and adhering to treatment.

At this point, it might be helpful to clear up a common source of confusion when comprehension is defined as a “process.” There is an equivocation between, on the one hand, “process” in the sense of something that can potentially grow and improve (as in “Don’t worry, as long as you keep reading and studying, your comprehension of philosophy will improve; it’s a slow process.”) and, on the other hand, “process” in the sense of an event or a series of events (e.g., reading comprehension as a covert process that accompanies outward reading behaviors). Comprehension, as we have seen, can come in degrees and improve with time, so in the first sense we can say, if we like, that comprehension is a process. However, it is not a process in the second sense for, as we have seen, it is not the name of any event nor series of events (our later discussion of the adverbial features of the concept will also help with this point).

The fact that comprehension can improve with time is sometimes put forward as an objection to the claim that comprehension is an achievement concept. To cure, the objection goes, is definitely an achievement, for either you cured someone, or you did not, but this all-or-none reasoning does not apply to the case of comprehension. However, the objection is misguided. Some achievement concepts are “all or none” (e.g., to win), whereas others admit degrees (e.g., to improve). They can also be different in various other aspects, but what holds them together as a category is that they help us speak, not of our actions, but of their results. A good test of an achievement concept is if it makes sense to say “I ____ed but with no success.” Independently of admitting degrees and nuances, comprehending is not something we do in the same sense as we read, study, run, or talk. It is a result of things we do, and if someone said, “I understood the text but did not grasp a word of it,” we would certainly be in doubt as to what the person meant. The muddled sense of comprehension as a private event, ongoing internal process, or class of behaviors has its origin, at least partly, in the confusions described in the last paragraphs.

#### Adverbial **f**eatures

Comprehension often manifests itself in how someone goes about doing something. For example, let us suppose we are listening to young Zoe reading a storybook aloud. She puts the stress on the right words, pauses for suspense, and colors her reading with a rich emotional tone. Perhaps she uses different voices that bring out the characters’ personalities and feelings. Zoe’s comprehension, as it were, “shines through” her reading. The way she reads expresses her comprehension. Similarly, there are contexts where we say someone carries out an activity with discernment or someone’s sayings or doings show deep or shallow comprehension of a subject.

Again, this tempts us to see comprehension as an event that is separate from the thing done. For, the reasoning goes, if, for example, Jonathan reads *with* comprehension, then comprehension must be something different from what he is doing, a separate process added to the reading (perhaps some accompanying covert events).

Layng, Sota, and Leon (2011), for example—although they did not, of course, defend that covert events are the cause of anything (as do those who champion a representationalist view of comprehension, e.g., Pearson & Cervetti, 2017)—did adopt the (in our view, mistaken) notion that comprehension must be, at least in part, some extra event that is added to what happens overtly. In this case, they posited covert verbal behavior that accompanies overt behavior in a step-by-step fashion, as becomes clear from their example of a young boy answering a multiple-choice comprehension question:

Let’s begin with John. As he moves his eyes along the text, he “hears” each word of text [*sic*] as it is read. “It was the first day of summer. Sam woke up early and ran outside, wondering what he should do first. Then he saw his new bike leaning against the tree. When Sam saw his new bike, he grinned.” But there is more. John “sees” Sam and what he is doing. When John reads that the “new bike [is] leaning against the tree,” he sees a sparkling bicycle, not an old, dirty one, and he sees it on an angle against a tree, not held up by a kickstand. John may also feel some of Sam’s excitement. Next, John reads the question, “How did Sam feel when he saw his new bicycle?” Again, he hears the words. He then reads the possible answers: “sad, happy, funny.” But this time he also hears himself say, “It wants me to guess at how Sam feels. I think funny sounds good.” He then puts a mark next to “funny.” (p. 3)

They later concluded, “What we just examined is an instance of what may be called reading comprehension” (p. 4). They then went on to look at the environmental variables involved, but the point here is that comprehension itself, in their foundational article, where they laid out the conceptual basis of their investigation, was explicitly identified with covert behavior that accompanies overt activity—that is, as a kind of parallel process.

But we might ask, is this not correct? After all, how could somebody’s activity show or express something else (comprehension), unless the two were separate? Ryle (1949/2009) helped us escape this confusion when he clarified the logic of expressions similar to “reading with comprehension” by explaining their adverbial function. To say that I am driving carefully, for example, is not to say that I am doing two things, driving and taking care. It is to say that I am driving in particular ways that are consistent with traffic rules, coherent with what is happening around me and avoiding accidents. This adverbial function is quite common when speaking of human behavior and does not befuddle us in other cases at all—for example, when we say that Mary sings with passion, Lola plans holiday trips with precision, and Veronica handles finances with great responsibility.

How do these features of the concept of comprehension relate to more conventional behavioral terminology? Although the polymorphous, dispositional, and adverbial features of the concept of comprehension do not map onto behavioral-analytic terms on a one-to-one basis (otherwise, they would not enlighten us in any way), we believe that this kind of conceptual analysis is (a) compatible with behavioral-analytic terminology and (b) an important part of functional analysis. Our analysis of comprehension is a token, not of empirical investigation (obviously) nor of theoretical construction (Leon, Layng, & Sota, 2011; for examples of these in the field of reading comprehension, see Sota, Leon, & Layng, 2011), but of what Machado and Silva (2007) regarded as one of the three pillars of scientific activity: conceptual clarification (mathematization/theory and empirical investigation/experimentation being the other two). The analysis of the concept of comprehension does not tell us anything about how, for example, shared reading interventions might help a child gain better comprehension of a story (an empirical question; for a recent example, see Gentilini & Greer, 2020), nor does it consist of theoretical proposals (as when we introduce the notion of degrees of embeddedness of reinforcement later in this article). It does, however, help us achieve clarity in our empirical questions and coherence in our pretheoretical assumptions, and this has direct consequences for empirical questions and theorizing. For example, as we have seen, clarity about what counts as “comprehension” makes it less tempting to assume that it refers to covert verbal behavior of some kind or to a single operant class, and encourages us to look more carefully at how the concept translates into contingencies of reinforcement in different human settings, as we will exemplify next.

### Two Applications of the Functional Approach: Legal and Narrative Texts

#### Legal Texts

The primary social function of legal systems, of which legal texts are a part, is to coercively control behavior that has been politically defined as socially undesirable. The expression “politically” emphasizes a common characteristic of modern legal systems: their dependence upon collectively binding decisions emanated from controlling agencies, such as a bill approved by Congress (Aguiar, 2017; Hart, 1994; Kelsen, 1960/1998; Skinner, 1953). The emphasis on coercive control acknowledges its predominance in law (Albert & Maluschke, 2013; Kelsen, 1960/1998; Schauer, 2015). Historically, the use of coercive control has been increasingly an exclusive prerogative of the State (Gilissen, 1979/2001; Weber, 1968).

Most legal texts, therefore, are a part of social contingencies that primarily establish certain behaviors as warranting punishment. They often either signal consequences for behavior or modify the signaling properties of other stimuli. For example, a bill on domestic violence is meant to signal punishment for potential offenders in a similar way as a warning given by an authority would. In some ways, it is as if the potential perpetrator were being given a warning by a person: “If you are violent toward your family, you will suffer consequences.” The text is not just a neutral description, but is meant to function already as one of the devices that suppress domestic violence. However, it will only do this if some conditions are in place.

First, mentions of sanctions in the text must have acquired aversive properties for the reader. The mention of prison, for example, may have acquired aversive functions through experiences such as witnessing the shame and hardship of family members of people in trouble with the law, seeing movies or documentaries depicting violence in prisons, transfer of functions from related expressions such as “confinement” and “suffering,” and personal experiences of loss of freedom, such as long hospitalizations or quarantine.

Second, in order for the text to have a deterring effect, sanctions must have been applied contingently to behavior. This will not be the case, for example, when threats of punishment for undesirable behavior are seldom carried out or, conversely, when people suffer aversive conditions independently of complying with the law (as when members of a community repeatedly experience injustice in the form of arbitrary violence or autocratic use of authority). The two conditions described so far show that the text’s function as a deterrent depends on previous learning experiences (it is a higher order behavioral process).

The third condition for legal texts to produce their intended effects on readers is their coherence with the wider contingencies of which they are a part. Just as a face-to-face threat will have no effect if it is clear that there is no way it can effectively be carried out, the legal text cannot function as a threat against wrongdoings if other environmental events signal a low probability of sanctions. In the case of the bill on domestic violence, some examples of environmental cues that can undermine its effects are the pervasiveness of offenses (as when there is a general social tolerance for domestic violence), social isolation of the victim and perpetrator (e.g., due to social-distancing measures), and dependency of the victim (e.g., financial or emotional).

Additionally, legal texts enter different behavioral relations for different actors. In our example, the bill on domestic violence signals, for the police officer, what she is authorized to do when notified, as well as what she is obliged to do (her duty to act against crime). At least for standard cases, it is as if she were receiving face-to-face authorization or instruction to act in a certain way, given certain states of affairs. For the victim, it might change the signaling function of the violence itself, for example, from something to be endured as part of the relationship and hidden from others, to an occasion for seeking help. It might also signal a lower probability of aversive consequences for countercontrol (Skinner, 1974), for example, by signaling the guarantee of financial aid, shelter, and protection for the victim and those in her care. For a neighbor or friend, it might signal a decrease in the probability of punishment for stepping in, offering help, or calling the police. For an attorney, the bill might signal that quoting it in her petition might boost the probability of reinforcement by increasing (prosecutors) or decreasing (defense attorneys) the chances that the judge or court will decide in favor of a given sanction (Aguiar, 2017). For each of the previous cases, the same restrictions apply that were mentioned earlier: The inducing functions of the text must have been established in the reader’s history, and its potential effects will depend on interactions with other environmental cues.

In each of these cases, we see that the text does not work on its own but only as it is embedded in wider social contingencies. What is sometimes referred to as “the correct interpretation” of a given text is related to the functions the text has in typical contexts for individuals with typical learning histories. Because we must inevitably differentiate between correct and incorrect interpretations of texts in many educational, professional, and research contexts, it might be illuminating to make explicit such typicality assumptions.

### Narrative Texts

One of the most admired aspects of narrative texts is their potential to inspire us to imagine other lives, times, and places and to evoke behavior that takes us well beyond the text, awakening vivid images of other worlds (Palmer, 2018). Narrative, it is said, is a privileged pathway into possible realities (e.g., Bruner, 2009) and a booster of our empathy (e.g., see Critchfield, 2018, for a functional account of literary empathy and the role of emotions in readers’ reactions to literary texts; Keen, 2006).

When we read, we sometimes say it is *as if* we could see a scene or a character. We might have imagined a character’s voice in a novel and then be sharply disappointed when we hear the actor in a movie based on the book. How is this possible? When we imagine Macbeth hallucinating a bloody dagger, are we looking at a scene that we have created, as we might look at a picture that we have drawn?

As we argued in our discussion of *as-if* patterns, this notion is muddled: “As if I were seeing” is not a (mysterious) kind of seeing. Ryle (1949/2009) illustrated this point with the example of a play. We see a murder onstage. But a murder onstage is not a kind of murder; on the contrary, it is not a murder at all. It is the staging of a murder, the pretending of a murder. For this to be possible, of course, actors and spectators alike must know what a murder is. Ryle said the staged murder is “parasitic” on real murders—that is, *as-if* murders are logically of a higher order than murders.

Similarly, when we “conjure images” of a story, we are not seeing anything, but behaving (in some ways) as if we were. As we argued earlier in the section on *as-if* patterns of behavior, it is because of the things we feel and are able to do in spite of the absence of any images or beyond those we have at hand (e.g., illustrations) that we say we are imagining scenes in a story. As Skinner (1963) put it, “The contingencies arranged by the verbal environment may set up self-descriptive responses describing the behavior of seeing even when the thing seen is not present” (p. 954).

Imagining stories, characters, and scenes is of a higher order (logically and developmentally) than experiences with nonimagined ones. A child cannot act as if she were facing a cat in a hat (or a hat-shaped cat, or any other fantastic being combining hats and cats) if she has no acquaintance with cats, nor with hats, and this is probably one of the reasons (though certainly not the only one, cf. Nikolajeva & Scott, 2001/2013; Sipe & Pantaleo, 2008) why we illustrate children’s books.

At any rate, so-called mental imagery is not as closely tied, conceptually speaking, to narrative comprehension as is commonly held. The fact that we, at first thought, tend to overrate its role probably has to do with the common but confusing conflation of imagination and so-called mental imagery (cf. Bennett & Hacker, 2003; Tanney, 2015). When we speak of imagining in the context of story comprehension, we are not always, nor even mainly, speaking of so-called mental imagery. To imagine a story is, in many cases, equivalent to understanding it. As Bennett and Hacker (2003) pointed out, to be able to imagine Othello’s rage and jealousy is simply to be able to conceive it (although not necessarily to sympathize with it).

In this second sense, to say that I can imagine a character or scene is equivalent to saying that my behavior is under the control of the relevant thematic units in the story (we will have more to say on thematic units in a moment). For this control to happen, I must have the relevant learning history (referred to as “background knowledge” or “world knowledge” in the comprehension literature). If I cannot imagine Romeo’s love for Juliet, say, because I come from a place where romantic love does not exist, nor anything like it, then this is equivalent to saying that the thematic unit of romantic love does not exert the relevant control over my behavior. For example, the scene at the balcony will not induce in me the same emotions as it will in someone from a culture where romantic love is central. The thematic unit of romantic love will also not combine with that of the enmity between the Capulets and Montagues in its effects on my behavior as it will for those acquainted with romantic love, for whom this combined control might occasion, for example, dreadful anticipation of tragedy.^5^ The ability to imagine Romeo and Juliet’s love is thus not equivalent to engaging in imagery-like behavior. The latter may or may not be one of the behaviors evoked by the thematic unit, but it is neither a necessary nor a sufficient condition for saying the reader can imagine Romeo and Juliet’s love for each other.

Far from affecting an isolated episode of our behavior, a failure of control by one thematic unit, such as in our Romeo and Juliet example, can jeopardize control by other important thematic units and thus affect comprehension of the whole story. This is probably true of many other kinds of texts as well, but it is certainly and centrally true in the case of narratives, because of two central and complementary characteristics of stories (Flores, Pires, & Souza, 2014; Flores, Rogoski, & Nolasco, 2020): (a) a relatively high independence of thematic units from specific formal divisions of the text and (b) how the thematic units are related to each other in their evoking and reinforcing effects. Let us explain each point.

The first point is that, in narratives, thematic units are rarely traceable to particular bits of a text (a feature often ignored in comprehension assessments and interventions, e.g., when students are instructed to locate exactly where they found the answer to a comprehension question). For example, Lady Macbeth’s ambition, a thematic unit, is not identical to any particular dialogue or act. It is “weaved” into the narrative and has no point-to-point correspondence with any formal subdivisions of the play. Thematic units in narratives can be explicit or merely suggested and can be instantiated not only by story events, dialogues, and descriptions, but also by text structure, repetition of words or expressions, metaphors, and so on.

The second point is that thematic units in narratives are very interdependent in their behavioral functions. An example is the well-known dramatic principle known as Chekhov’s gun, mentioned by Barthes and Duisit (1975). The principle holds that nothing in a narrative should be gratuitous (“If there is mention of a gun, it should be fired.”). When the gun makes its appearance, this prompts us to behave (perhaps expect a tragedy at some point). If the story then surprises us with a character’s vacillation to fire or with a happy ending, the surprise itself depends on our previous reaction to when the gun was mentioned. When reading Nabokov’s *Lolita*, told from the point of view of a very unreliable narrator, we at first take his version at face value. We are soon led to reconsider our first impressions. As we advance in the novel, what we read differentially reinforces previous verbal responses and occasions new ones. We regret having let ourselves be misled and even charmed by such a despicable character.

In sum, narratives offer not only occasions for behavior but also a network of arranged contingencies where one thematic unit induces behavior, for which another unit acts as a consequence (e.g., we thought the butler was the murderer, but a new scene makes this implausible and sets the occasion for additional hypothesizing to be reinforced).^6^ We say it is a network and not a chain because a thematic unit might also change the effect of a previous unit on our verbal behavior (e.g., we come to recognize it as a well-planned “false lead”).^7^ This possibility is especially favored by narratives because, as we said before, the same thematic units tend to reappear (varying in their form) throughout the story, prompting us to respond to them again, but each time under a new combined control of other new thematic units. This recursive movement in the reader’s behavior may be why creative writing teachers insist there must be a balance between what is said and what is left unsaid in narratives. If you say everything, the reader has no opportunity to behave—the story is too predictable. If you leave too many gaps, there is insufficient differential reinforcement for the reader’s responses—the plot is patchy and unconvincing.

As with any other texts, narratives will only exert their unique effects if their recursive evoking and reinforcing functions have been established through the practices of a verbal community. These practices usually begin early. They might include hearing family members tell their personal experiences and being encouraged, from early childhood, to tell our own (“What did you do with Grandma today? Oh, did you bake a cake? I’ll bet it was yummy! What kind of cake was it?”; e.g., Haden, Haine, & Fivush, 1997; Minami, 2001). They might also include hearing traditional or religious oral narratives, looking at the pictures in books and comics, engaging in pretend play, watching movies and cartoons, participating in story time at school or at the library, and a myriad of other, interconnected practices (there will, of course, be variations, culturally and historically, and the relative weights of different activities on reading behaviors are subjects for empirical enquiry).

These social contingencies establish the typical signaling and reinforcing functions of narrative thematic units. An example is a child’s exposure to multiple variations of fairy tales and traditional stories. With time, she comes to react to the famous “Once upon a time . . .” in conventional ways. This learning history becomes evident if the storyteller jokingly fails to follow the established conventions (e.g., “Once upon a time, a fish was swimming with a whale, the whale swallowed the fish, and that’s the end of the tale.”). The child laughs (or not). She may protest, ask for a “real story,” or seem bewildered. Something is not right; certain previously experienced patterns have been violated—the storyteller has broken a rule. In other words, the child’s behavior as a listener, occasioned by the familiar “Once upon a time . . . ,” was not followed by the usual social consequences (see Hineline, 2018, for an analysis of other behavioral processes potentially at work during storytelling, including joint attention and tracking repertoires).

These repeating narrative practices eventually crystallize into narrative genres. The genre, in a general sense, is a summarized description, on the cultural level, of how a text is typically used and the wider social contingencies it is usually a part of. Structuralists have shown that such contingencies form recognizable patterns, a well-known example being Propp’s (1928/1971) analysis of the structure of fairy tales.^8^ As our reinforcement histories with different narrative genres evolve, certain recurring story structures and tropes begin to function as thematic units in their own right, affecting the discriminative function of other thematic units as soon as we start reading (e.g., compare how a reader might approach a sci-fi novel vs. a historical one).

### A Preliminary Framework for the Classification of Reader–Text Contingencies

We hope that the previous exercise of applying our general analysis to legal and narrative texts has given concreteness to our argument that it is not advantageous to consider texts as having a univocal discriminative function. This does not necessarily imply that we are condemned to work on a case-by-case basis. As we have seen, the roles texts play in different social environments tend to be reflected in certain regularities in the functional and structural features of those texts.^9^ At this point, therefore, an attempt at systematization may be in order. We propose a first approximation to a functional classification of reader–text contingencies, based on antecedents (setting) and consequences.

First, we apply the notion of *openness of setting* to reader–text contingencies. More open settings are those where reinforcement is arranged for a greater variety of behaviors, as opposed to more closed settings, where we do not have much choice on how to behave (cf. Foxall, 1998, 2016). We thus consider reader–text contingencies to include a behavioral setting that can be more or less open in this sense. When reading a poem, for example, the verbal community usually arranges reinforcement for a wider range of responses than when reading an informative paragraph about the honeybee waggle dance. The poem, that is, offers a more open setting than the scientific text.

When applying the notion of openness of setting, however, we must keep in mind what we discussed earlier: Texts do not acquire their effects on our behavior on their own nor through mere textual training, but through their roles in our broader human environments and practices. A prayer book to be followed during church service constitutes a relatively closed setting as a part of a ritualized activity, with preestablished and unchanging proceedings. A typical storybook for young children, in contrast, forms a relatively open setting, with attractive illustrations and exciting story events, as parts of activities in which spontaneity and playfulness are often encouraged (e.g., parent–child shared reading and “story time” in community libraries).

Let us now look at the consequences for the reader’s behaviors. We have shown how, for some texts, an important part of reinforcement is arranged to happen as a result of continuing to read the same text. We have discussed how narratives, for example, allow for later thematic units in the text to differentially reinforce behavior occasioned by earlier ones, and for recursive changes to take place in the functions of thematic units as the reader advances. For many other kinds of texts, on the contrary, reinforcement for reading happens predominantly “outside” the text. Reinforcement for following an instruction manual or applying a legal definition, for example, depends largely on events beyond the text. For lack of a better expression, we refer to this as the degree of *embeddedness of reinforcement*. It expresses the degree to which reinforcement for the reader’s behavior is arranged to happen through further reading of the same text as opposed to reinforcement arranged for nonreading responses (although related to reading, e.g., assembling a piece of furniture by reading instructions).

**Fig. 1:**
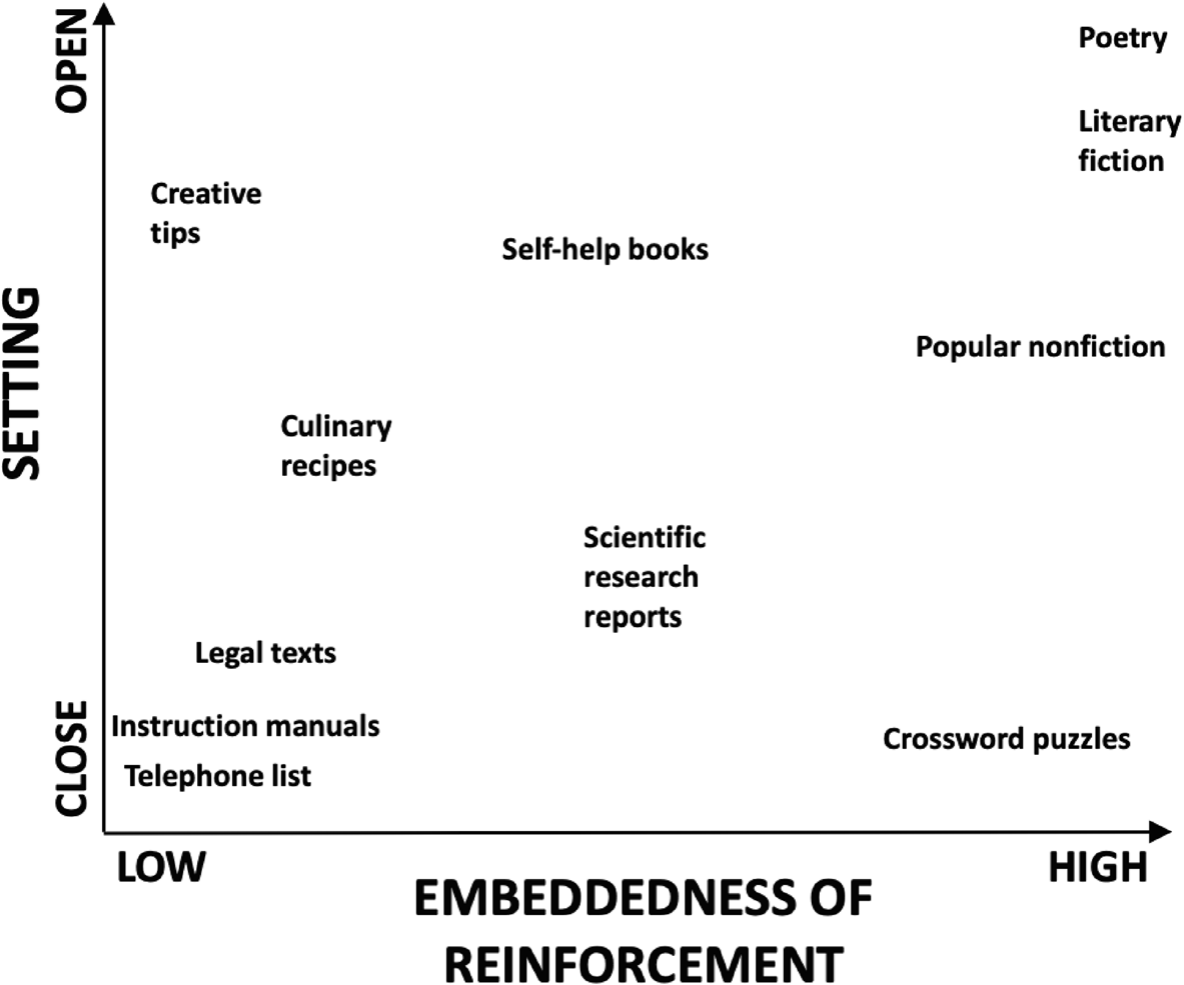
Examples of combinations of openness of setting and embeddedness of reinforcement

Figure 1 shows some examples of how texts can be classified according to these two dimensions (openness of setting and embeddedness of reinforcement). Some texts constitute very open settings and have high embeddedness—a modern poem, for example. Instruction manuals are useful as closed settings and have low embeddedness, telephone lists even more so.^10^

Texts with exceptionally closed settings and high embeddedness are relatively hard to find, for a simple reason. Where there is high embeddedness of reinforcement, there must be something to differentially reinforce, so behavior must vary (the setting cannot be too closed). But even in high-embeddedness texts, there is room for variation in the openness of setting. An example comes up when we compare commercial page-turners, on the one hand, and literary fiction, on the other. Allowing for exceptions in both directions, commercial “best seller” type novels tend to have more predictable text structures, whereas literary fiction often challenges readers with less structured patterns and gives them more responsibility in the process.

As for texts with low embeddedness, self-help books usually have more open settings than instruction manuals, common law more than statutory law, and jazz scores more than classical scores.

## Conclusion

We hope to have clarified some common confusions and introduced a conceptual framework that may be helpful in the construction of empirical and theoretical questions related to reading. Taking into account the polymorphous property of comprehension might make us more attentive to the typical activities and learning histories involved in the particular kind of text we are working with when studying reading and reading comprehension.

If our account of comprehension is correct, inquiries into the wider contingencies that surround and are embedded in texts will be more fruitful than the standard approach of regarding reading comprehension as a general ability.^11^ Consequently, we might also become weary of assuming that there is such a thing as a typical, neutral, or central kind of text from which others are derivations.^12^ The framework we have proposed for classifying texts according to the openness of setting and embeddedness of reinforcement can help us avoid the fallacy of the universal text, while still allowing for continuities and comparisons. We leave it to the readers to imagine interesting experimental manipulations of these dimensions.

The aforementioned are only examples of the upshots of our analysis. We wish to leave the setting of this article open enough to provoke further debate. We also hope it contains enough embeddedness of reinforcement to have kept the reader with us up to this point.

## Footnotes

1 To avoid excessive scare quotes throughout the text, we will dispense with them when using vernacular terms related to comprehension and understanding. We are not adopting the terms but referring to the discourse that includes them (in other words, we are describing the contingencies and practices in vigor when they are employed).

2 Skinner (1957) used the term “thematic” in opposition to “formal.” Whereas formal verbal relations take place when there are physical similarities or conventional point-to-point connections between environmental events and verbal behavior, thematic control describes relationships not defined by point-to-point links between a stimulus and response. Thematically controlled verbal expression is usually more open-ended and less constrained by formal properties of the stimulus (cf. Michael, Palmer, & Sundberg, 2011).

3 Relational frame theory has expanded the variety of pairings (e.g., Barnes-Holmes, Finn, McEnteggart, & Barnes-Holmes, 2018; Hayes, Barnes-Holmes, & Roche, 2001), but the grounding of language on stimulus associations (in terms of derived relational networks) has remained unchanged.

4 For an analysis of the difficulties involved in equating such correlations with representations, see Bennet and Hacker (2003), especially their analysis of the notion of brain maps.

5 Compare Bartlett (1932) for a classical experimental demonstration of how the failure of control by culturally specific thematic units affects the behavior evoked by narratives.

6 Grant (2005) interpreted this as an aversive contingency (the situation of suspense) from which we seek to “escape” by continuing to read and reaching a resolution. We see two potential problems in this view: First, the use of “aversive” is metaphorical, for if the encounter with the climax of a narrative (e.g., in a detective story) were nonmetaphorically aversive, we would avoid contact with it by not picking up the book in the first place. Second, the notion is too restrictive—not all narratives follow the tension-relief or suspense-resolution sequence.

7 After elaborating this functional interpretation of narrative as a set of programmed contingencies, we became aware of a similar, independent conclusion by Hineline (2018) based on the establishing function of suspense and the reinforcing properties of resolution. Hineline’s metaphor of puzzle pieces and more general emphasis on story coherence are compatible with our proposal of degrees of embedded reinforcement in different kinds of texts, explained further on.

8 A well-known exception is the modern literary novel, perhaps because rapid changes in social and cultural contingencies are reflected in equally rapid changes in forms, tropes, and metaphors (cf. Bakhtin, 1941/1981).

9 Hineline (2018) noted, in the context of fictional narratives, that this is a topic “that does not easily fit into the behavior-analytic rubric, *nor into the functional/structural distinction* [emphasis added]” (p. 473).

10 Incidentally, a recent series of poems generated by Internet users implicitly played with this kind of contrast by putting together verses from the results of Google searches (examples can be seen at http://www.googlepoetics.com). The reader was led to consider the results of Google searches as if they were poems. In other words, verbal behaviors typically induced by the open settings and high embeddedness of poems were generalized to closed-setting, low-embeddedness textual contexts (an invitation to see the poetic in the nonpoetic).

11 We are not denying that there might be generalization from one setting to the other; on the contrary, it is the acknowledgment of the polymorphous nature of comprehension that will help us not take generalization for granted and thus investigate it.

12 Coincidentally or not, the supposedly prototypical texts used in our labs are often watered-down versions of the informative texts that we scientists are so familiar with.
